# Research hotspots and frotiers of stem cells in stroke: A bibliometric analysis from 2004 to 2022

**DOI:** 10.3389/fphar.2023.1111815

**Published:** 2023-03-03

**Authors:** Qi Zhang, Yuting Zeng, Shuqi Zheng, Ling Chen, Haining Liu, Hui Chen, Xiaofeng Zhang, Jihua Zou, Xiaoyan Zheng, Yantong Wan, Guozhi Huang, Qing Zeng

**Affiliations:** ^1^ Department of Rehabilitation Medicine, Zhujiang Hospital, Southern Medical University, Guangzhou, China; ^2^ School of Rehabilitation Medicine, Southern Medical University, Guangzhou, China; ^3^ Faculty of Health and Social Sciences, The Hong Kong Polytechnic University, Hong Kong, China; ^4^ Guangdong Provincial Key Laboratory of Proteomics, Department of Pathophysiology, School of Basic Medical Sciences, Southern Medical University, Guangzhou, China

**Keywords:** stem cells, stroke, citespace, VOSviewer, web of science, bibliometrics

## Abstract

**Background:** Stroke is one of the leading causes of mortality and permanent disability worldwide. However, the current stroke treatment has a limited effect. Therefore, a new treatment is urgently needed. Stem cell therapy is a cutting-edge treatment for stroke patients. This study aimed to gain better understanding of global stem cell trends in stroke *via* a bibliometric analysis.

**Methods:** We used the Web of Science Core Collection to search pertinent articles about stem cells in stroke published between 2004 and 2022. Analysis was conducted using CiteSpace, VOSviewer, and the R package “bibliometrix” to identify publication outputs, countries/regions, institutions, authors/co-cited authors, journals/co-cited journals, co-cited references, and keywords.

**Results:** A total of 6,703 publications were included in the bibliometric analysis. The total number of citations significantly and rapidly increased between 2004 and 2022, with the most pronounced growth pattern observed in the period of 2008–2009. In terms of authoritarian countries, the USA had the most publications among the countries. As for institutions and authors, the most prolific institution was the University of South Florida, followed by Oakland University and then Shanghai Jiao Tong University, and Chopp, M. and Borlongan, Cesario V, had the most output among the authors. Regarding the journals, *Cell Transplantation* had the highest publication, followed by *Brain Research*. As for references, “Mesenchymal stem cells as trophic mediators” was the most frequently cited (2,082), and the article entitled *Neuronal replacement from endogenous precursors in the adult brain after stroke* had the strongest burstiness (strength = 81.35). Emerging hot words in the past decade included “adhesion molecule,” “mesenchymal stromal cell,” “extracellular vesicle,” “pluripotent stem cells,” “signaling pathway,” “plasticity,” and “exosomes.”

**Conclusion:** Between 2004 and 2022, the terms “neurogenesis,” “angiogenesis,” “mesenchymal stem cells,” “extracellular vesicle,” “exosomes,” “inflammation,” and “oxidative stress” have emerged as the hot research areas for research on stem cells in stroke. Although stem cells exert a number of positive effects, the main mechanisms for mitigating the damage caused by stroke are still unknown. Clinical challenges may include complicating factors that can affect the efficacy of stem cell therapy, which are worth a deep exploration.

## 1 Introduction

Stroke is currently the third major cause of adult disability and the second leading cause of mortality worldwide (Owolabi et al., 2022). Following a stroke, the brain may be damaged by neuronal apoptosis, oxidative stress, and cytotoxic cascade reactions (Kuriakose and Xiao, 2020). Stem cell therapy, an emerging treatment option for stroke, has the potential to improve neurological outcomes and functions by promoting neurogenesis, reducing oxidative stress, and decreasing cytotoxicity (Zhao et al., 2022). At present, many stem cell types have been shown to be effective in treating stroke, such as pluripotent stem cells (Duan et al., 2021), neural stem cells (NSCs) (Zhang et al., 2017), embryonic stem cells (Xia et al., 2021), and mesenchymal stem cells (MSCs) (Bedini et al., 2018). In addition, many studies have demonstrated the capacity of these stem cells for brain rewiring (Pluchino and Peruzzotti-Jametti, 2013), neoangiogenesis (Nam et al., 2015), inflammatory inhibition (He et al., 2021a), and nerve regeneration (Ould-Brahim et al., 2018). Scholars have published a plethora of basic research and clinical trials on stem cell therapy in stroke, but new and comprehensive quantitative evidence to support the direction and research hotspots in this field is limited. Thus, it is necessary to review the development of research on stem cells in stroke from 2004 to 2022 and to present an objective analysis based on data from publications as a foundation for future study.

Bibliometric analysis is a statistical method for forecasting knowledge structure and hotspots within a certain field of study through visual representations (Ninkov et al., 2022). By reading this kind of study, readers may be able to obtain quantitative information on how journals are distributed by nation, organization, author, and journal in a specific field (Zhang L. et al., 2022). Bibliometric analysis provides unambiguous insights into many medical areas (Kokol et al., 2021). However, bibliometric studies conducted in the field of stem cells in stroke are scarce. As a result of the dramatic increase in stem cell research and publications over the past several years, the necessity to integrate and renew research data in a bibliometric analysis on stem cells in stroke has arisen.

As a response to the paucity of quantitative analysis of research regarding stem cells in stroke, the present study acquired global scientific research on stem cells in stroke between 2004 and 2022 with quantitative information on the publication outputs, countries/regions, institutions, authors/co-cited authors, journals/co-cited journals, co-cited and burst references, keywords, and burst keywords. This study aimed to highlight hotspots for study in this area by synthesizing research direction and emergent themes from these investigations.

## 2 Methods

### 2.1 Search strategy and data acquisition

The Web of Science (WoS) contains 20,000 reputable academic publications that span 250 different fields worldwide (Zhong and Lin, 2022). Other academic researchers in the field of bibliography have used the WoS as the most trustworthy data source for data extraction in bibliometric analysis (Dong et al., 2022).

**FIGURE 1:**
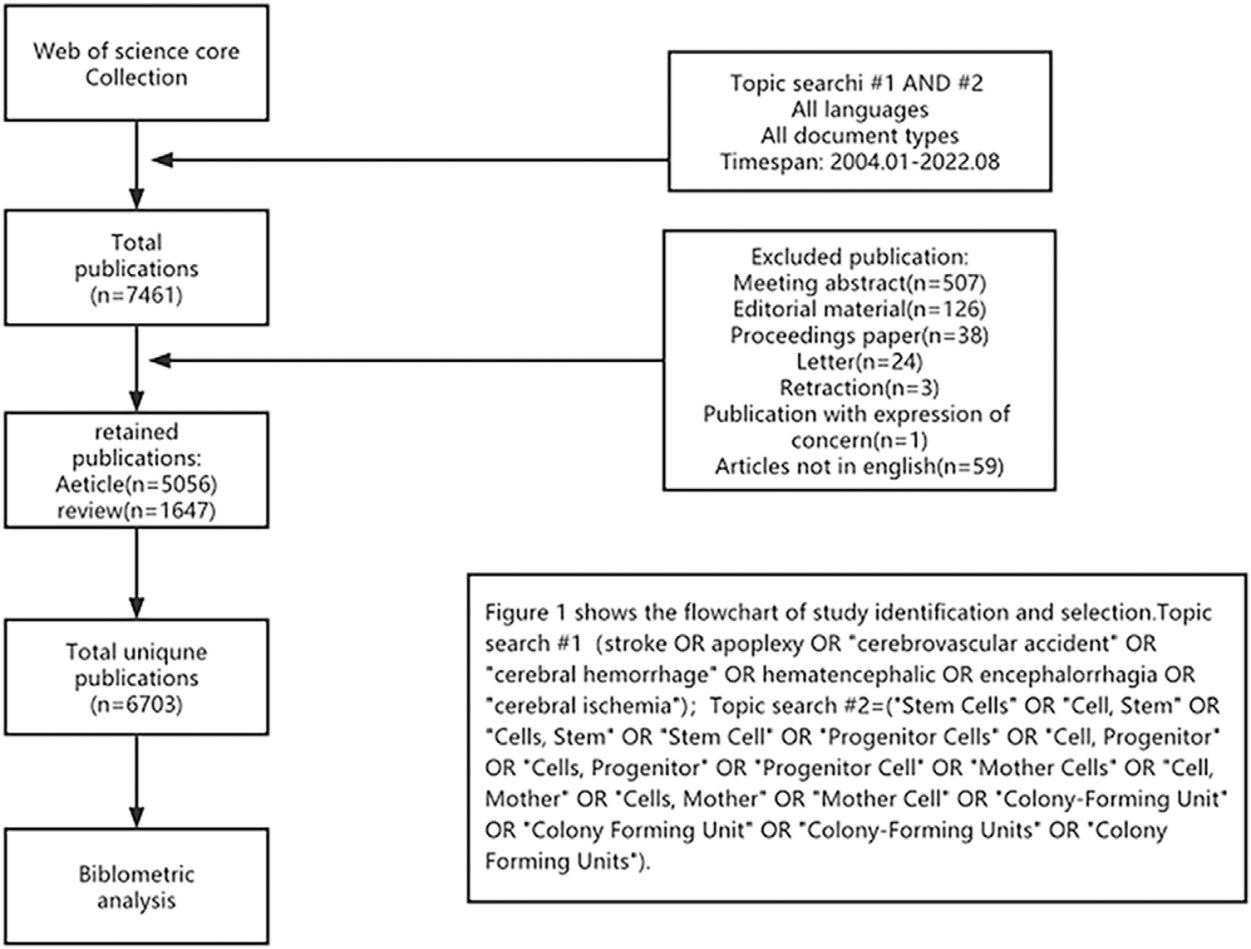
Flow chart of the screening process for research on stem cells in stroke.

We conducted a comprehensive literature search using the Web of Science Core Collection (WoSCC) database from 1 January 2004 to 11 August 2022. To obtain as comprehensive and accurate results as possible, the search strategies we used were TS=(stroke OR apoplexy OR “cerebrovascular accident” OR “cerebral hemorrhage” OR hematencephalic OR encephalorrhagia OR “cerebral ischemia”) AND TS=(“Stem Cells” OR “Cell, Stem” OR “Cells, Stem” OR “Stem Cell” OR “Progenitor Cells” OR “Cell, Progenitor” OR “Cells, Progenitor” OR “Progenitor Cell” OR “Mother Cells” OR “Cell, Mother” OR “Cells, Mother” OR “Mother Cell” OR “Colony-Forming Unit” OR “Colony Forming Unit” OR “Colony-Forming Units” OR “Colony Forming Units”). Only articles and reviews were included. Furthermore, letters, commentaries, meeting abstracts, and other types of documents were excluded. Finally, 6,703 records were included for analysis. The specific literature screening process is presented in Figure 1.

### 2.2 Data analysis

The original data downloaded from the WoSCC were firstly imported into Microsoft Excel 2016, and then two authors (QZh and YZ) independently screened the final included articles and collected all data from the final papers that were included, such as titles, authors, keywords, institutions, countries/regions, citations, journals, and publication dates. Subsequently, the processed data was imported to VOSviewer (version 1.6.15), CiteSpace (version 5.8), and R package “bibliometrix” for bibliometric analysis.

CiteSpace is a bibliometric software that enables the analysis and visualization of trends and patterns in a research area (Pan et al., 2018; J; Zhang and Lin, 2022). It also creates a knowledge map of connected fields, clearly presents the panoramic information of a particular knowledge field, and identifies the critical studies, hot research, tendency, and frontiers of a specific scientific field using a variety of dynamic network analysis techniques (Godfrey et al., 2018; Y; Chen, Lin, and Zhuang, 2022). CiteSpace was used in this study to conduct co-occurrence and cluster analyses of authors, research institutions, nations, and discipline features. The parameters of CiteSpace were set as follows: in the Time Slicing column time settings 2004.01–2022.08, each year is a time slice.

The Leiden University Center for Science and Technology Studies (CWTS) created VOSviewer, a software for creating and analyzing bibliometric networks (Netherlands). VOSviewer can extract bibliographic networks (co-authorship, co-occurrence, and citation-based) from bibliographic data (Lin, Chen, and Chen, 2020; Moral-Muñoz et al., 2020; Luo and Lin, 2021). In this study, co-occurrence and cluster analyses of authors, research institutions, countries, and discipline features were conducted using CiteSpace.

Bibliometrix (https://www.bibliometrix.org) is an open-source R package developed by Dr. Massimo Aria and Corrado Cuccurullo from Naples University in Italy. It is capable of conducting comprehensive bibliometric and scientometric analyses (Moral-Muñoz et al., 2020). In this study, bibliometrix was used to create a global distribution network of articles on stem cells in stroke and to analyze the thematic evolution of those publications (Aria and Cuccurullo, 2017).

## 3 Results

### 3.1 Temporal trend of publication outputs

**FIGURE 2:**
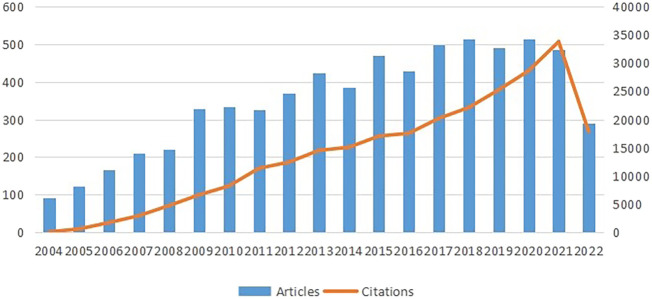
Trends of annual publications on research of stem cells in stroke. The data for 2022 is not complete.

As can be seen from Figure 2, the histogram and curves exhibit two trends: the total number of papers published and citations per year. Both trends grow throughout time, illustrating the direction in which research in this area is moving. The number of citations significantly and rapidly increased between 2004 and 2021, suggesting that research on stem cells in stroke has attracted interest. From 2004 to 2007, the number of literature grew rapidly, and in 2009, it dramatically increased. However, the number of articles remained relatively stable from 2010 to 2017. The year 2020 had the most number of publications in recent years, which peaked at 515. Although the data for 2022 have not yet been completed, it is predicted that they will exhibit a moderate trend compared with those in the previous year.

### 3.2 Contributions of countries/regions

**FIGURE 3:**
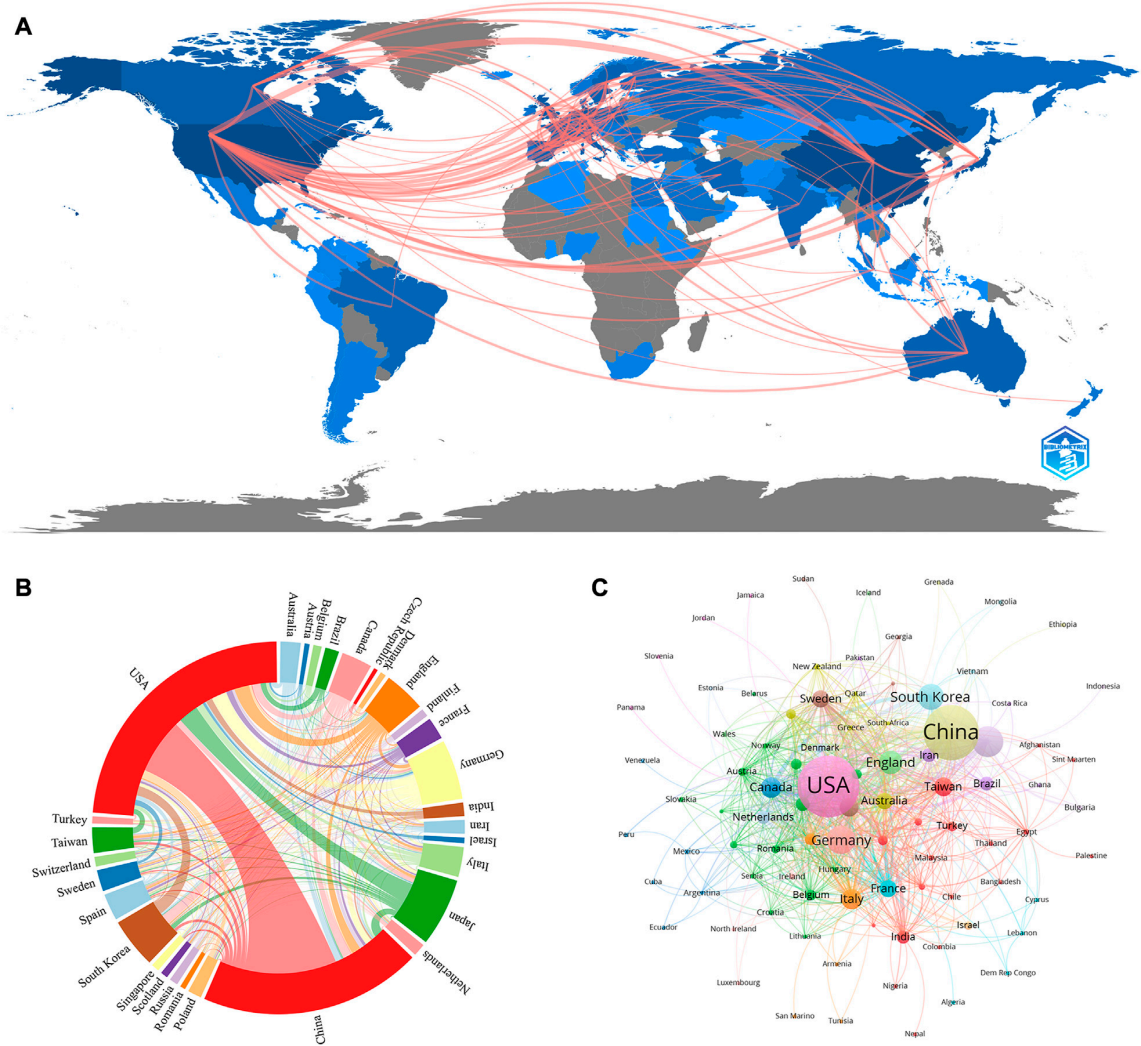
**(A)** Country/region collaboration map. The Darker blues indicated higher collaboration rates, the wider the link line, the higher the rate of collaboration between the two countries. **(B)** Distribution of countries/regions in the field of stem cells in stroke. The size of the circle represents the number of articles issued by countries or regions, and the line means cooperation among countries or regions **(C)** Distribution and international cooperation of countries that are involved in stem cells in stroke research. The thickness of the line reflects the frequency of the cooperation. The thicker the line, the stronger the cooperation.

As for the geographical distribution, 6,703 documents were published from 94 different countries and regions; Table 1 presents the top 10 countries/regions in this category. As can be seen from Figure 3B and Supplementary Figure S1, we also used VOSviewer for the visual analysis of countries or regions. The USA published the most papers (1,555papers, 25.61%), followed by China (738 papers, 12.15%) and then Japan (714papers, 11.76%), indicating that these three countries play a crucial role in this field. The quantity and connections among publications in each nation were then used to create a collaborative network (Figure 3A). A country collaboration analysis was conducted on the 30 countries with the highest number of publications in this area (Figure 3C). According to the total link strength, the top five countries/regions were the USA, China, Japan, Germany, and South Korea.

**TABLE 1:** Top 10 countries/regions with highest publications on stem cells in stroke.

Rank	Countries/Regions	Total link strength	Count (%)
1	United States	1220	1555 (25.61%)
2	China	585	738 (12.15%)
3	Japan	497	714 (11.76%)
4	Germany	426	342 (5.63%)
5	South Korea	303	280 (4.61%)
6	England	271	271 (4.46%)
7	Canada	244	249 (4.10%)
8	Italy	243	224 (3.69%)
9	Spain	238	220 (3.62%)
10	Taiwan	216	216 (3.56%)

### 3.3 Contributions of institutions

**FIGURE 4:**
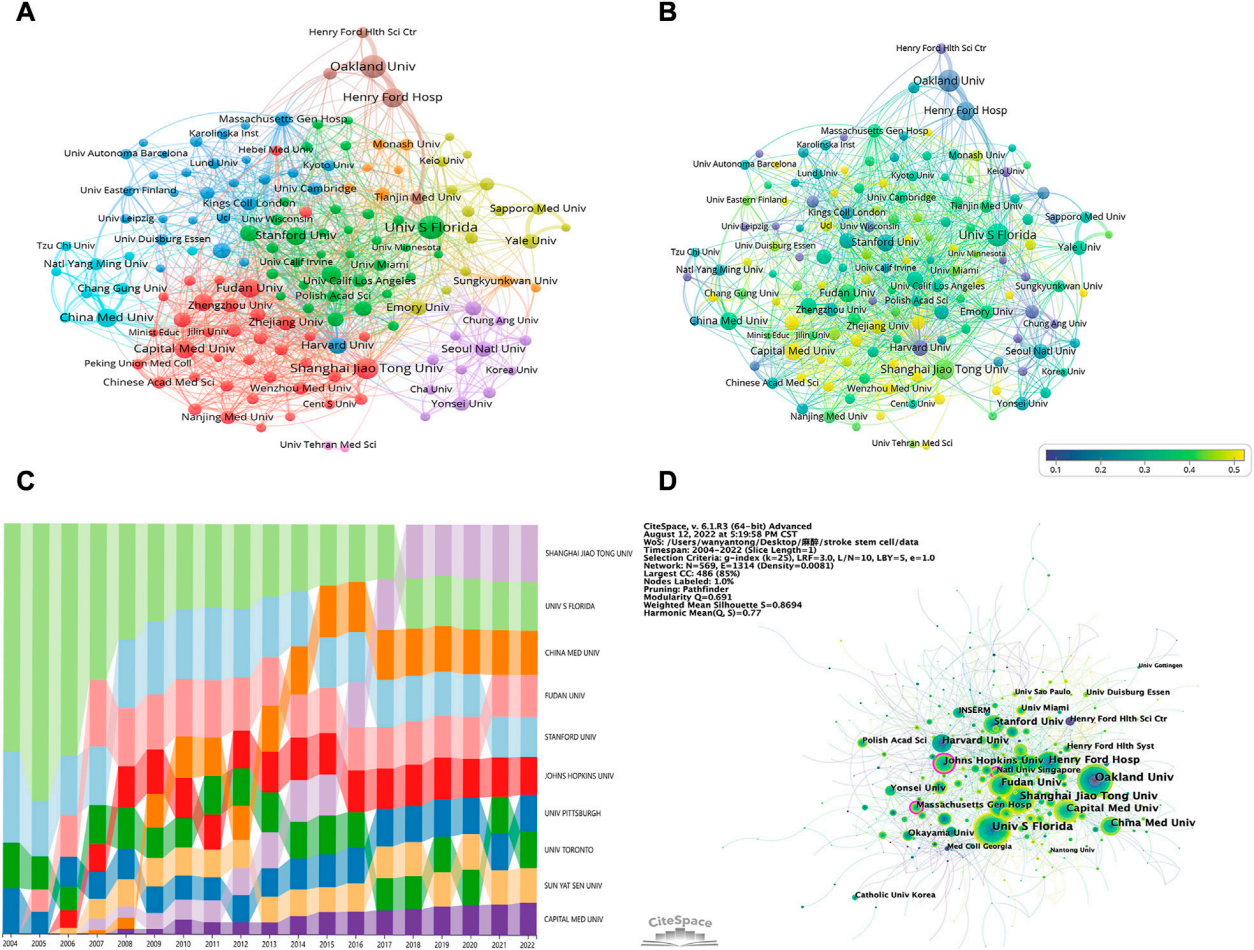
**(A)** Institutions clustering analysis. The node size signifies the number of publications of institutions, and the thickness of the line signifies the strength of cooperation among institutions; node colors signify different clusters. **(B)** Timeline visualization of collaboration among institutions. The blue to yellow gradient represents the number of articles published in the last 5 years as a percentage of the total number of articles published by the organization **(C)** Trends of publications in this field by different institutions over time. Different colors represent different institutions, the high and low positions represent the ranking of the institution’s publication quantity, and the width represents the proportion of the institution’s publication quantity among the top 10 institutions’ publications of the year **(D)** Institutions related to stem cells in stroke. The larger the circle, the more articles the institution posts. The purple circle represents centrality, and the line means cooperation among institutions.

In Table 2, the top 10 institutions with the highest productivity are ranked by their productivity. China and the USA were the only two countries where the highest productivity were located. The most prolific institution was the University of South Florida (164 publications, 2.70%), followed by the Oakland University (151 publications, 2.49%) and then the Shanghai Jiao Tong University (129 publications, 2.12%). The clustering analysis of institutions is presented in Figure 4A. A tight and continuous interaction between institutions can also be observed. Among them, the institutions with more collaborations were Oakland University, Henry Ford Hospital, and Henry Ford Health Science Center, followed by Sapporo Medical University and Yale University. In Figure 4B, we analyzed the data of articles published in the last 5 years using VOSviewer. For instance, Harvard University started research in the field earlier and had published significantly more articles in the past than it had recently. Contrarily, Capital Medical University entered the field later and has recently published a higher number of articles. As can be seen from the cluster analysis figure, the red and light blue circles indicate mainly Chinese institutions. Combined with the visual timeline, it can be seen that Chinese institutions are predominantly yellow, indicating that they entered the field late or have recently published a high number of articles.

**TABLE 2:** Top 10 institutions related to stem cells in stroke.

Rank	Institution	Count (%)	Country	Institution	Total link strength
1	Univ S Florida	164 (2.70%)	United States	Oakland Univ	182
2	Oakland Univ	151 (2.49%)	United States	Henry Ford Hosp	133
3	Shanghai Jiao Tong Univ	129 (2.12%)	China	China Med Univ	114
4	Henry Ford Hosp	107 (1.76%)	United States	Harvard Univ	102
5	Fudan Univ	102 (1.68%)	China	Univ S Florida	101
6	China Med Univ	97 (1.60%)	China	Shanghai Jiao Tong Univ	91
7	Capital Med Univ	96 (1.58%)	China	Massachusetts Gen Hosp	88
8	Stanford Univ	91 (1.50%)	United States	Seoul Natl Univ	87
9	Sun Yat Sen Univ	85 (1.40%)	China	Univ Pittsburgh	79
10	Johns Hopkins Univ	79 (1.35%)	United States	Univ British Columbia	79

Figure 4C presents the publication trend in this field by different institutions over time. The University of South Florida accounted for more than half publications from 2004 to 2006 and then gradually declined; however, the University of South Florida remained in the leading position, demonstrating the remarkable contributions of this institution to this sector. The rest of the institutions exhibited an upward trend in the publication quantity. Interestingly, Shanghai Jiao Tong University has made the most significant progress. Since 2018, Shanghai Jiao Tong University has, unsurprisingly, ranked first among the 10 institutions in terms of publication ratio. We also used CiteSpace for the visual analysis of institution clusters and marked them with keywords (Figure 4D). Consequently, the Johns Hopkins University, Massachusetts General Hospital, and National University of Singapore exhibited high centrality. The largest cluster in Supplementary Figure S1 was designated “ischemic stroke” (cluster #0), indicating that various institutions are most concerned with this word. It was followed by “clinical trials” (cluster #1), “primate” (cluster #2), and “extracellular matrix” (cluster #3), respectively. Other important clusters were “mesenchymal stem cell,” “neurological function,” and “hypothermia.”

### 3.4 Authors and Co-cited authors

**FIGURE 5:**
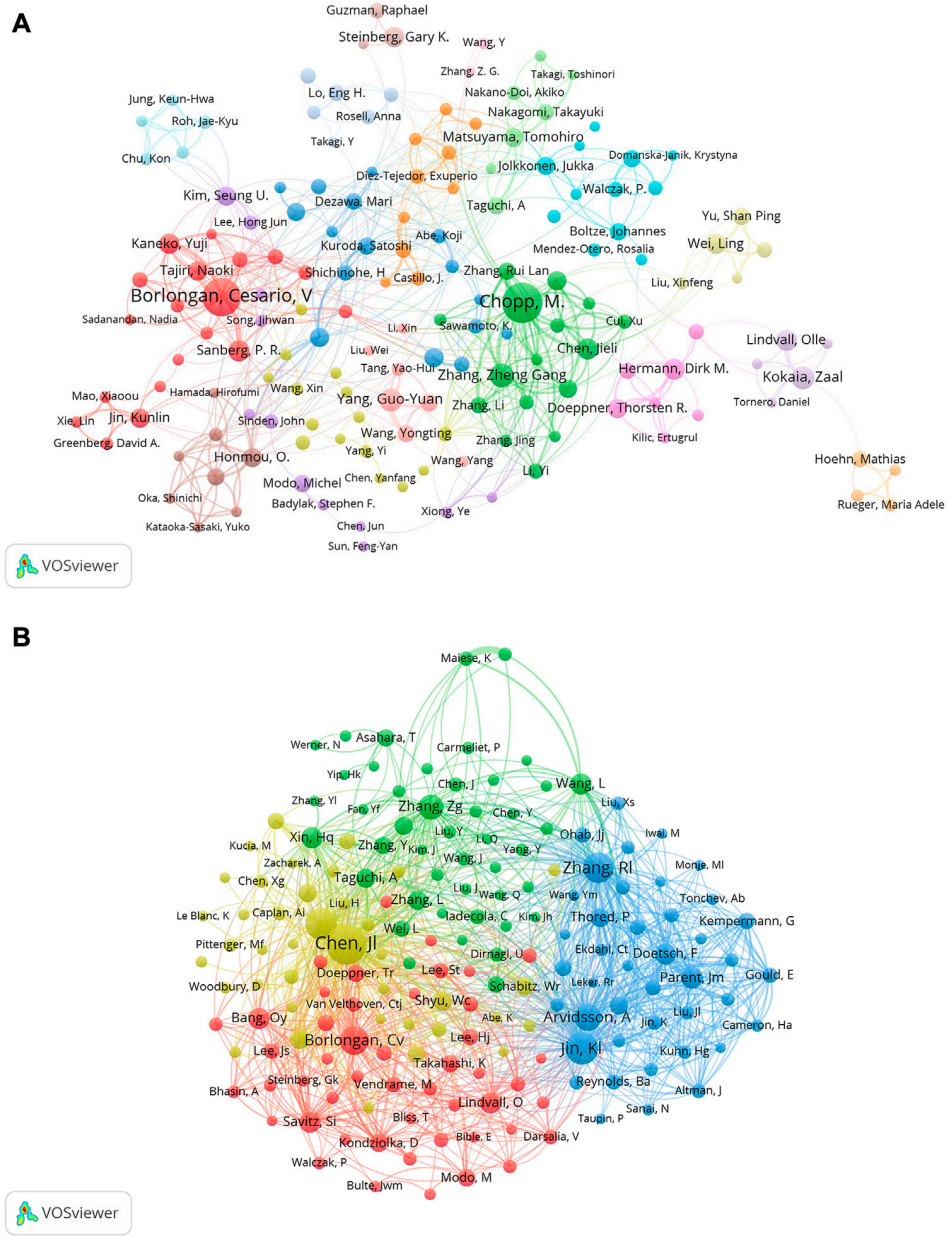
**(A)** Co-author related to stem cells in stroke. Each circle represents an author, the lines between the circles signify connections between the authors, and the networks of connections in different colors signify cooperative clusters among different authors **(B)** Co-cited-author clustering analysis.

Approximately 174 writers contributed to a total of 6,703 articles. The most prolific author was Chopp, M. who produced 160 articles (2.63%), closely followed by Borlongan, Cesario, V who produced 143 publications (2.35%). Three authors published 50 and more articles (Zhang Zheng Gang, Yang Guo-Yuan, and Kokaia Zaal), and five authors published 40 and more articles (Hermann Dirk M. Chen Jieli, Lindvall Olle, Sanberg P. R. and Kaneko Yuji) (Table 3). Information on co-authors and co-cited authors was also analyzed using VOSviewer (Figures 5A, B). Figure 5A shows that there are primarily two research teams involved in author collaboration, led by Chopp, M. and Borlongan, Cesario, V, who frequently and closely collaborate with other authors. Co-cited authors are those who have had two or more of their names concurrently mentioned in one or more subsequent articles and who are therefore considered to have a co-citation connection. A total of more than 1000 citations have been received by the top six authors among the top 10 co-cited writers (Table 3). The most frequently referenced author was Chen Jl. (n = 2,214), followed by Jin, Kl (n = 1,473), Li, Y (n = 1,367), Zhang, RI (n = 1,313), Borlongan, Cv (n = 1,144), and Avidsson, A (n = 1,003).

**TABLE 3:** Top 10 authors and co-cited authors related to stem cells in stroke.

Rank	Author	Count (%)	Total link strength	Author	Co-citations	Total link strength
1	Chopp, M.	160 (2.63%)	392	Chen, Jl	2214	36563
2	Borlongan, Cesario, V	143 (2.35%)	232	Jin, Kl	1473	29645
3	Zhang, Zheng Gang	57 (0.94%)	169	Li, Y	1367	22711
4	Yang, Guo-Yuan	52 (0.86%)	89	Zhang, Rl	1313	25956
5	Kokaia, Zaal	50 (0.82%)	77	Borlongan, Cv	1144	18474
6	Hermann, Dirk M.	45 (0.74%)	86	Arvidsson, A	1003	17582
7	Chen, Jieli	42 (0.69%)	148	Zhang, Zg	879	15015
8	Lindvall, Olle	42 (0.69%)	69	Parent, Jm	701	13781
9	Sanberg, P. R.	42 (0.69%)	64	Lindvall, O	646	10025
10	Kaneko, Yuji	40 (0.66%)	112	Savitz, Si	643	12778

### 3.5 Journals and Co-cited academic journals

We found that 252 journals published 6,703 papers regarding stem cells in stroke. As can be seen from Table 4, it is clear that the journal *Cell Transplantation* has the most papers (172, 2.83%), followed by *Brain Research* (160, 2.63%). Among the top 10 journals, *Stroke* (10.17) has the greatest impact factor (IF). The number of times the top 10 most co-cited journals are cited determines their influence. As presented in Table 4, the publication with the most citations is *Stroke* (19,776), indicating that it has a significant impact in this category, followed by the *Journal of Neuroscience* (14,061) and *Proceedings of the National Academy of Sciences of the United States of Americ*a (11,769).

**TABLE 4:** Top 10 journals and co-cited journals related to stem cells in stroke.

Rank	Journal	Count (%)	IF(JCR 2020)	JCR Quartile	Co-cited-journal	Citations	IF(JCR 2020)	JCR Quartile
1	Cell Transplantation	172 (2.83%)	4.139	Q3	Stroke	19776	10.17	Q1
2	Brain Research	160 (2.63%)	3.61	Q3	J Neurosci	14061	6.709	Q1
3	Stroke	158 (2.60%)	10.17	Q1	P Natl Acad Sci Usa	11769	12.779	Q1
4	Plos One	147 (2.42%)	3.752	Q2	J Cerebr Blood F Met	10862	6.96	Q1
5	Journal Of Cerebral Blood Flow And Metabolism	137 (2.26%)	6.96	Q1	Brain Res	8227	3.61	Q3
6	Neural Regeneration Research	129 (2.12%)	6.058	Q2	Nature	7386	69.504	Q1
7	International Journal Of Molecular Sciences	113 (1.86%)	6.208	Q1	Plos One	7295	3.752	Q2
8	Neuroscience	103 (1.70%)	3.708	Q3	Stem Cells	6787	5.845	Q1
9	Experimental Neurology	102 (1.68%)	5.62	Q2	Exp Neurol	6630	5.62	Q2
10	Stem Cell Research and Therapy	95 (1.56%)	8.098	Q1	Science	6519	63.798	Q1

**FIGURE 6:**
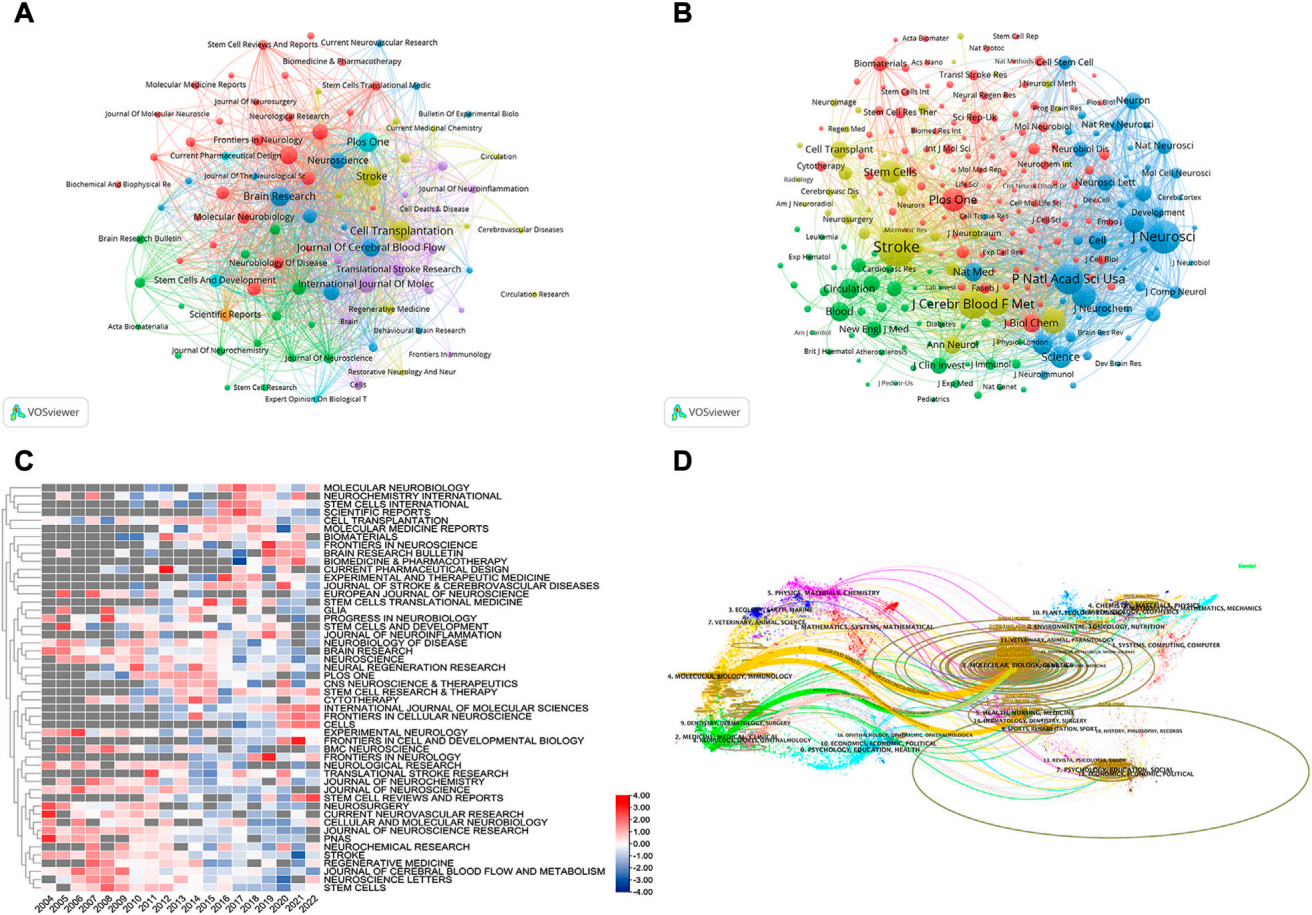
**(A)** Co-journal clustering analysis. Each circle represents a journal, the size of the circle depends on the strength of the connection, the number of citations, and so on. And, the color of the circle on behalf of the cluster to which it belongs, different clusters are represented by different colors. **(B)** Co-cited-journal clustering analysis **(C)** Journals heat map on stem cells in stroke. Each line was compared with each other. The value in the box is the number of articles published in the journal divided by the number of articles published in the year, verbalizing the current year’s popularity of the journal, The redder the color verbalizes the hotter journal in the year, so it was called the heat map **(D)** The dual-map overlay of journal publishing research. Citing journals on the left and cited journals on the right, and the curve is the citation line, which completely shows the context of the citation, the more papers the journal publishes, the longer the vertical axis of the ellipse; the more authors they are, the longer the horizontal axis of the ellipse.

Using VOSviewer, we conducted a visual analysis of the published journals and obtained details about journal collaboration through Figures 6A, B. We could see that the journals of *Stroke*, *Archives of Physical Medicine and Rehabilitation*, and *Neurorehabilitation and Neural Repair* had more times of co-citation and greater influence. We also conducted comparative analysis of the journals’ popularity, as presented in Figure 6C. Through this heat map, we can understand the change in the research direction and emphasis in this field and grasp the development trends. We found that in recent years, the popularity of *NEUROSURGERY*, *CURRENT NEUROVASCULAR RESEARCH*, and *PANS* had gradually decreased, whereas that of *CELLS*, *INTERNATIONAL JOURNAL OF MOLECULAR SCIENCES*, and *FRONTIERS IN CELLULAR NEUROSCIENCE* had gradually increased. One of the interesting things about *STEM CELL REVIEWS AND REPORTS* is that its popularity had declined year by year, but in the last 2 years, it had returned to its level in 2004. Furthermore, on the dual-map overlay of journal publishing research (Figure 6D), we found four citation paths (colors orange, pink, and green), demonstrating that the studies published in molecular/biology/genetics journals and health/nursing/medicine journals were mainly cited by the studies published in molecular/biology/immunology, medicine/medical/clinical, and neurology/sports/ophthalmology journals.

### 3.6 Co-cited reference and reference bursts

The top 15 documents that were cited the most often out of the 6703 retrieved are listed in Table 5. *Mesenchymal stem cells as trophic mediators* was the most frequently cited (2,082), which is a review of studies on the applications of adult marrow-derived MCSs. It was followed by *Concise review: Mesenchymal stem cells: Their phenotype, differentiation capacity, immunological features, and potential for homing* (1,725) and then *Adult mesenchymal stem cells for tissue engineering versus regenerative medicine* (1,378).

**TABLE 5:** Top 15 co-cited references related to stem cells in stroke.

Rank	Author	Article title	Source title	Cited	Year	DOI
1	Caplan, AI, et al.	Mesenchymal stem cells as trophic mediators	JOURNAL OF CELLULAR BIOCHEMISTRY	2082	2006	10.1002/jcb.20886
2	Chamberlain, G, et al.	Concise review: Mesenchymal stem cells: Their phenotype, differentiation capacity, immunological features, and potential for homing	STEM CELLS	1725	2007	10.1634/stemcells.2007-0197
3	Caplan, AI	Adult mesenchymal stem cells for tissue engineering *versus* regenerative medicine	JOURNAL OF CELLULAR PHYSIOLOGY	1378	2007	10.1002/jcp.21200
4	Moskowitz, MA, et al.	The Science of Stroke: Mechanisms in Search of Treatments	NEURON	1303	2010	10.1016/j.neuron.2010.07.002
5	Langhorne, P, et al.	Stroke Care 2 Stroke rehabilitation	LANCET	1290	2011	10.1016/S0140-6736 (11)60325-5
6	Schmidt-Lucke, C, et al.	Reduced number of circulating endothelial progenitor cells predicts future cardiovascular events - Proof of concept for the clinical importance of endogenous vascular repair	CIRCULATION	915	2005	10.1161/CIRCULATIONAHA.104.504,340
7	Imitola, J, et al.	Directed migration of neural stem cells to sites of CNS injury by the stromal cell-derived factor 1 alpha/CXC chemokine receptor 4 pathway	PROCEEDINGS OF THE NATIONAL ACADEMY OF SCIENCES OF THE UNITED STATES OF AMERICA	851	2004	10.1073/pnas.0408258102
8	Bang, OY, et al.	Autologous mesenchymal stem cell transplantation in stroke patients	ANNALS OF NEUROLOGY	838	2005	10.1002/ana.20501
9	Abrous, DN, et al.	Adult neurogenesis: From precursors to network and physiology	PHYSIOLOGICAL REVIEWS	750	2005	10.1152/physrev.00055.2003
10	Lalu, MM, et al.	Safety of Cell Therapy with Mesenchymal Stromal Cells (SafeCell): A Systematic Review and Meta-Analysis of Clinical Trials	PLOS ONE	728	2012	10.1371/journal.pone.0047559
11	Falk, E	Pathogenesis of atherosclerosis	JOURNAL OF THE AMERICAN COLLEGE OF CARDIOLOGY	715	2006	10.1016/j.jacc. 2005.09.068
12	Dutta, P, et al.	Myocardial infarction accelerates atherosclerosis	NATURE	688	2012	10.1038/nature11260
13	Amariglio, N, et al.	Donor-Derived Brain Tumor Following Neural Stem Cell Transplantation in an Ataxia Telangiectasia Patient	PLOS MEDICINE	671	2009	10.1371/journal.pmed.1000029
14	Ohab, JJ, et al.	A neurovascular niche for neurogenesis after stroke	JOURNAL OF NEUROSCIENCE	661	2006	10.1523/JNEUROSCI.4323-06.2006
15	Lochhead, JJ, et al.	Intranasal delivery of biologics to the central nervous system	ADVANCED DRUG DELIVERY REVIEWS	628	2012	10.1016/j.addr. 2011.11.002

**FIGURE 7:**
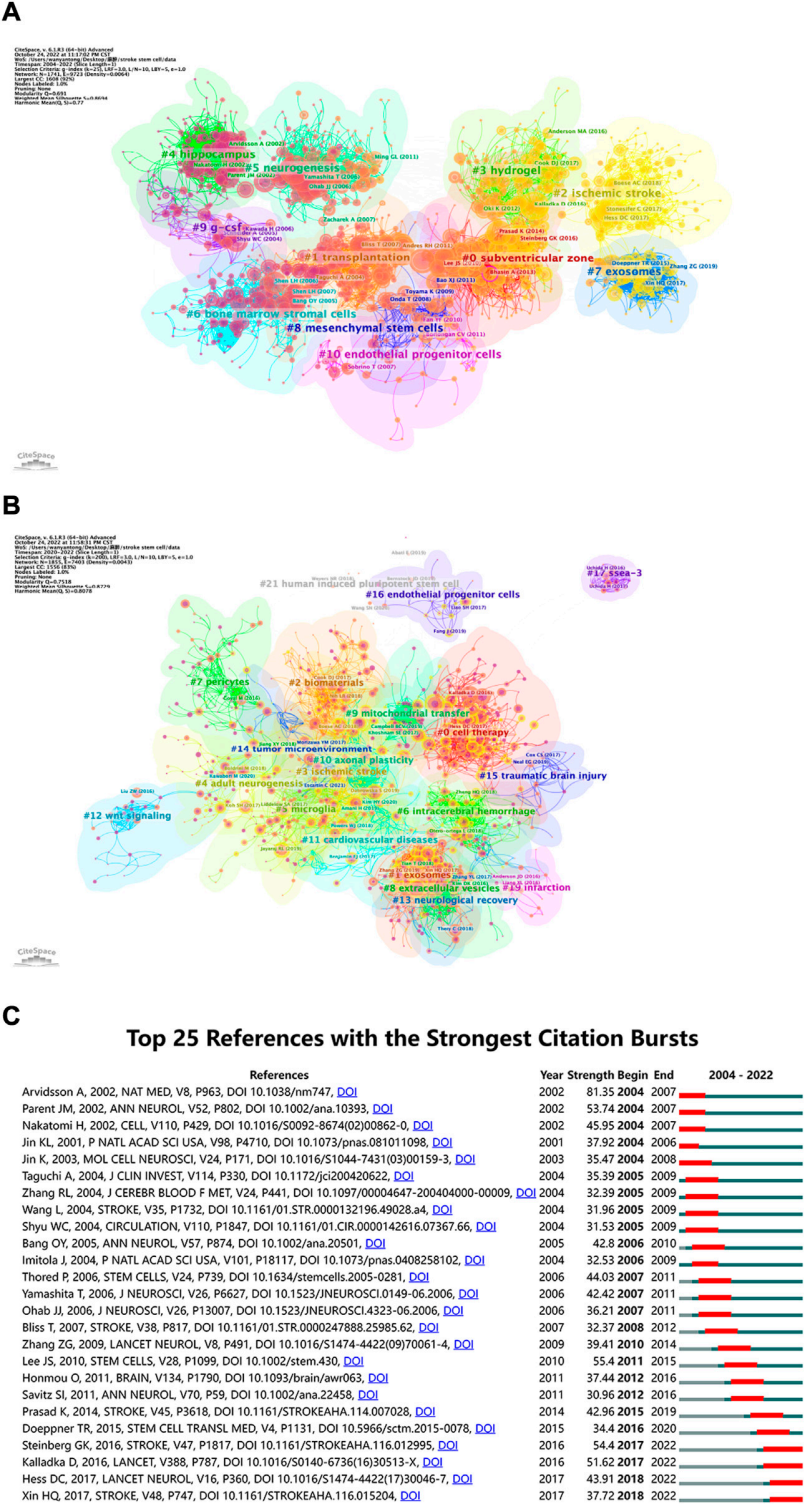
**(A)** Co-cited references related to stem cells in stroke. The circles represent the number of co-citations, the purple circle represents centrality, the thickness of connection indicates the cooperation degree, and the purple to yellow gradient represents the time from the past to the present. **(B)** Cluster view of references in stem cells in stroke research in recent 3 years. The larger the circle, the more times the corresponding paper has been cited in the last 3 years **(C)** CiteSpace visualization map of top 25 references with the strongest citation bursts involved in stem cells in stroke. The blue line represents the time interval. The blue line represents the time interval. The time in which a reference was found to have a burst is displayed by a red line, indicating the first year and the last year of the duration of the burst.

When two or more articles are simultaneously cited in the same article, the relationship between the simultaneously cited articles is referred to as co-citation. As presented in Figure 7A, we also used the CiteSpace clustering function to create a visual map to cluster the co-citation literature, and the collected literature was separated into 11 clusters *via* cluster analysis. Each cluster was intimately connected to the others and worked together in specific areas. The weighted mean silhouette and modularity Q were 0.8694 and 0.691, respectively, demonstrating the stability, believability, and persuasiveness of the clustering structure. Figure 7A illustrates the time dimension by the color of circle changing from purple to yellow, which also shows the change in the direction and concentration of the research. While the “hippocampus” cluster had received more attention in the past, researchers recently turned their attention to “exosomes,” “hydrogels,” and “ischemic stroke.” The name of the biggest cluster was “subventricular zone” (cluster #0), which was followed by “transplantation” (cluster #1), “ischemic stroke” (cluster #2), and “hydrogel” (cluster #3). The “hippocampus,” “neurogenesis,” “bone marrow stromal cells (BMSCs),” and “exosomes” clusters were other significant groups that may have represented a turning point in some sense.

As shown in Figure 7B, we created a visual map to cluster the cited literature over the last 3 years and divided them into 21 clusters through cluster analysis using CiteSpace. The weighted mean silhouette and modularity Q were 0.8729 and 0.7518, respectively, demonstrating the stability, believability, and persuasiveness of the clustering structure. The Figure 7B can help us get the latest research hotspots. The largest cluster was “cell treatment” (cluster #0), which was followed by “exosomes” (cluster #1), “biomaterials” (cluster #2), and “ischemic stroke” (cluster #3). Additional significant clusters were “adult neurogenesis,” “microglia,” “intracerebral hemorrhage,” and “pericytes.” Combined with Figure 7A, the “hippocampus” cluster has received more attention in the past, researchers have recently turned their attention to “exosomes,” “hydrogels,” and “ischemic stroke.” In Figure 7C, the top 25 references are listed in chronological order, which have the greatest burst intensity. References that receive several citations over a period of time are known as “citation burst” references. We set the time period in CiteSpace to 2004–2022 and still kept references with a burst termination date of 2022. *Neuronal replacement from endogenous antecedents in the adult brain following stroke* by Andreas et al. was published in *Nature Medicine* in 2002, and it had the strongest burstiness (strength = 81.35), occurring from 2004 to 2007 (Arvidsson et al., 2002). The advantages and disadvantages of the top 25 articles with the strongest citation bursts are summarized in Supplementary Table S1.

### 3.7 Key topics of research hotspots

**FIGURE 8:**
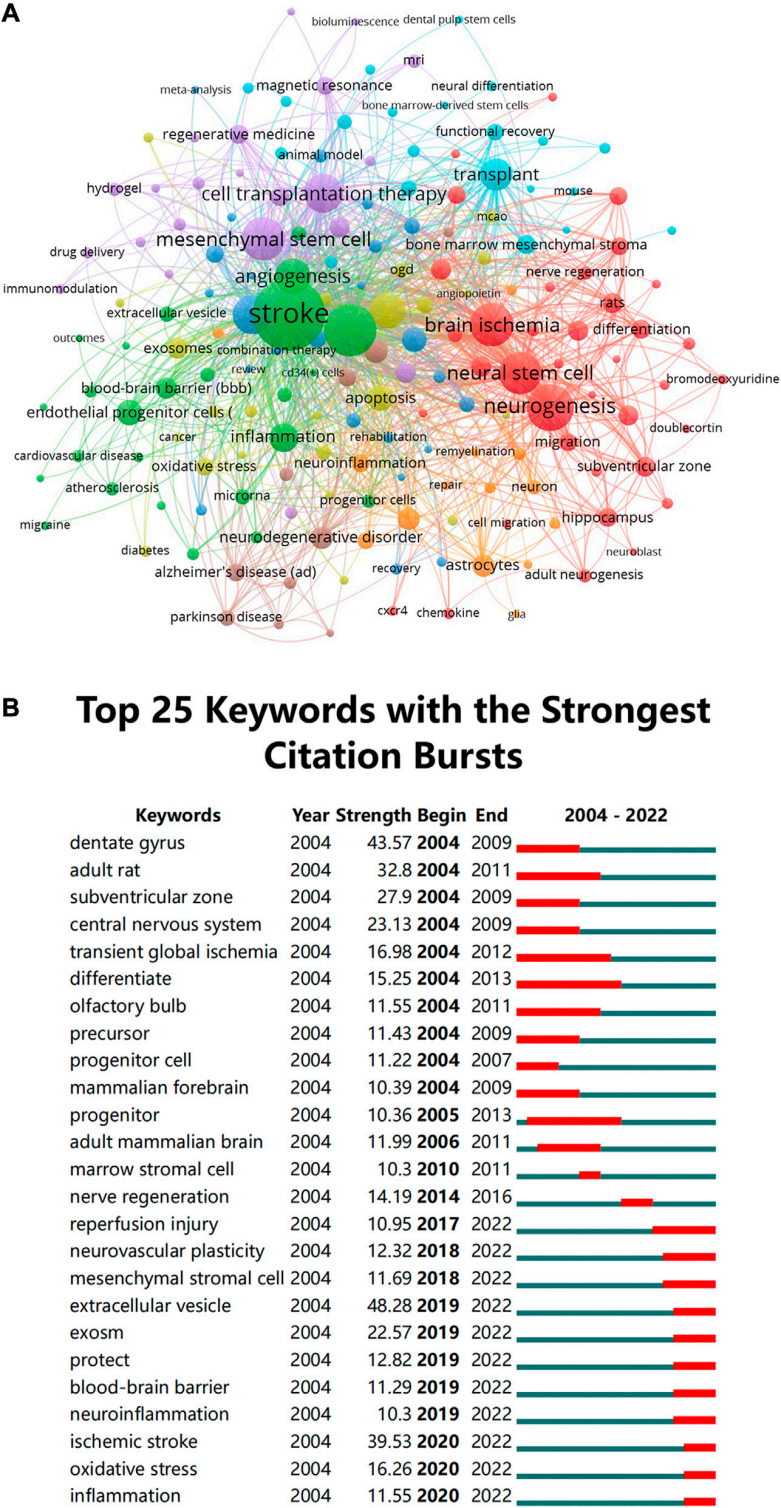
**(A)** Clustering of co-occurrence among keywords. The circles and labels in the figure constitute a unit, and the units of different colors constitute different clusters **(B)** CiteSpace visualization map of top 25 keywords with the strongest citation bursts involved in stem cells in stroke.

The use of cluster analysis to cluster the included keywords and summarize the study subjects might be helpful for relevant researchers in identifying popular topics and assisting scholars in better understanding current scientific concerns. We used VOSviewer to cluster the keywords into eight, as presented in Figure 8A: origin and behavior of stem cells (red), pathophysiological process of stroke (green), treatment of stroke and application of stem cells (purple), effects of stem cell therapy after stroke (light blue), cells that make up the central nervous system and the pathophysiological changes (orange), other diseases associated with stem cell therapy (brown), exosomes and mechanism of action (yellow), others (navy blue).

Meanwhile, we performed a series of keyword burst detections. To evaluate the development of stem cells in stroke research, researchers used a method named “keyword burst detection,” which is the recognition of phrases that often occur in a certain period of time. Table 6 demonstrates that terms with a high frequency in this study, aside from “stroke” (1538), include “ischemia” (801), “neurogenesis” (660), “brain ischemia” (553), “mesenchymal stem cell” (549), and “neural stem” (541). Prominent keywords were further analyzed. The top 25 keywords are listed Figure 8B in chronological order, which have the greatest burst intensity. It can be seen from the figure that the keywords that had exploded in the past 5 years mainly focused on “extracellular vesicles,” “inflammation regulation,” and “regulation of extracellular matrix.” A timeline analysis that visually depicts the research hotspots and development paths of stem cells in stroke at various stages from a temporal perspective is presented in Supplementary Figure S2. The keywords that have received extensive attention in this field appear early, for example, cerebral ischemia, regeneration, NSC, endothelial progenitor cells, apoptosis, and cell therapy. Emerging hot words in the past decade include adhesion molecule, mesenchymal stromal cell, extracellular vesicle, pluripotent stem cell, signaling pathway, plasticity, and exosomes.

**TABLE 6:** Top 20 keywords related to stem cells in stroke.

Rank	Keyword	Occurrences	Total link strength	Rank	Keyword	Occurrences	Total link strength
1	stroke	1538	4098	11	transplant	306	934
2	ischemia	801	2025	12	inflammation	259	804
3	neurogenesis	660	1878	13	endothelial progenitor cells (epcs)	204	436
4	brain ischemia	553	1381	14	apoptosis	191	554
5	mesenchymal stem cell	549	1307	15	stem cell	185	561
6	neural stem cell	541	1447	16	traumatic brain injury	161	490
7	stem cells	464	1351	17	neurodegenerative disorder	156	489
8	cell transplantation therapy	461	1327	18	microglia	150	444
9	angiogenesis	387	1157	19	astrocytes	144	429
10	neuronal protection	382	1087	20	blood-brain barrier (BBB)	138	391

## 4 Discussion

### 4.1 Global research trends of stem cells in stroke

This study aimed to conduct a bibliometric analysis of the last 18 years’ worth of research on stem cells in stroke. The citation count has been steadily increasing each year, with the 2008–2009 period showing the clearest growth pattern. Prior to 2008, an average of 150 papers per year were published in this field. From 2009 to 2022, the number of publications gradually increased, with an average of 350 papers published annually. The number of articles published in 2020 peaked at 515. These findings indicated that studies on stem cell therapy for stroke have gained increasing attention from researchers from all over the world in recent years, and the research area is currently in a steady developmental stage.

The top three authoritarian countries performing research on stem cells in stroke are the USA, China, and Japan. Four European nations, four Asian-Pacific nations, and two American nations make up the top ten nations. Furthermore, among the top 10 research institutions, five were American, and the remaining five were Chinese. We observed a close coordination between four nations, namely, Germany, Japan, China, and the United States. China also actively works with Japan, Canada, and England. There are some academic institutions that collaborate well with one another, such as Shanghai Jiao Tong University, Capital Medical University, and Harvard University. Although Oakland University ranked second in terms of paper publications, it was found to have limited collaborations with other universities. Such collaborations are detrimental for these universities in terms of long-term scholarly advancement. Consequently, it is indeed necessary that research organizations from many nations work closely together and communicate to collaboratively promote stem cell therapy for stroke.

As for the journals, those listed in Table 4 may be the core journals of the publication of research on stem cells in stroke. The most widely followed journal in this field of study is *Cell Transplantation* (IF = 4.139, Q3), with the majority of research on stem cell therapy published in this journal. *Stroke* (IF = 10.17, Q1) has the greatest IF, which also received the most citations (19,776 times). According to the Journal Citation Reports (2021 edition), five journals had IF values between 5 and 10 (*Journal of Cerebral Blood Flow And Metabolism*, *International Journal Of Molecular Sciences*, *Experimental Neurology*, *Stem Cell Research and Therapy*, and *Neural Regeneration Research*), four had an IF value between 3 and 5 (*Cell Transplantation*, *Brain Research*, *Plos One*, and *Neuroscience*), and no journal had an IF value below 3. These results indicated that most studies were published in these high-quality journals, and when researching on this topic, academics should concentrate on the content published in these journals. Most of the co-cited journals are high-impact Q1 journals, as can be seen from the list of co-cited journals. These journals support the investigation of stem cells in stroke and are undoubtedly of high quality. Furthermore, current research on stem cells in stroke is mostly published in journals pertaining to molecular biology, immunology, and medicine, followed by journals about neurology, sports, and ophthalmology. This indicates that basic research still constitutes the focus of the research and that the proportion of clinical studies is relatively low.

As for the authors, Chopp, M. (Oakland University, USA) is the most prolific, followed by Borlongan, Cesario, V (Stanford University, USA). They contributed to so many publications and were leaders in this field. Before 2015, Professor Chopp, M. and his team mainly focused on the role of stem cells in stroke, including BMSCs, neural progenitor cells, and human umbilical cord blood cells. In 2002, they found that after adult mouse suffered focal cerebral ischemia, endothelial progenitor cells generated from bone marrow contributed to brain neovascularization (Z. G. Zhang et al., 2002). Then in 2006, they demonstrated that intracarotid transplantation of BMSCs increased axon-myelin remodeling following stroke (L.H. Shen et al., 2006). Their experimental results in 2010 indicated that following a stroke in mice, MSC-mediated enhanced tPA activation in astrocytes encouraged neurite development (Xin et al., 2010). The next year, they published a study on how the production of astrocytic endogenous glial cell-generated neurotrophic factor was enhanced by BMSC implantation in the ischemic boundary area following stroke in adult rats (Shen et al., 2010). In the same year, they confirmed that the subventricular area has more progenitor cells dividing than normal due to the human umbilical cord tissue-derived cells (hUTCs) (Zhang et al., 2011) and had a neurorestorative effect (Ding et al., 2013). They proposed that the level of proinflammatory factors in the blood can be significantly reduced after hUTC transplantation (Bae et al., 2012). Furthermore, Chopp, M. discovered several chemicals that control stem cell migration, differentiation, and proliferation, such as specific miRNAs (X. S. Liu et al., 2013; Buller et al., 2012), atorvastatin (R. L. Zhang et al., 2012; J; Chen et al., 2008), angiopoietin 2 (X. S. Liu et al., 2009), and erythropoietin (L. Wang et al., 2008). These findings provided a solid theoretical basis for the rapid development of stem cells in stroke. Afterwards, Chopp, M. and his team shifted the attention to MSC-derived exosomes and described how they contribute to immunological reactivity, vascular remodeling, and brain regeneration during stroke recovery. In addition, they provided an overview of the potential and perspectives of stem cells in the fields of stroke and regenerative medicine, the links between stem cells and inflammatory factors for stroke rehabilitation, and the prospects of exosomes in the field of stroke. In the recent 2 years, Chopp et al. gradually focused on and studied the application of extracellular vesicles in neurological diseases, indicating that the extracellular vesicles may perhaps become a new research hotspot in this field.

The most commonly co-cited author is Chen, Jl. (citation = 2,214), followed by Jin, K.L. (citation = 1,473), and Li, Y. (citation = 1,367). In 2006, Chen, Jl. investigated the effects of giving human BMSCs (hBMSCs) intravenously after intracerebral hemorrhage (ICH) in rats and found that doing so dramatically improves neurological function (Seyfried et al., 2006). This paper provided the basis for the clinical investigation of BMSCs in ICH. The next year, Chen, Jl. demonstrated neurological recovery in rats intravenously injected with hBMSCs 1 month following a stroke (Shen et al., 2007b) and published the first 1-year follow-up report of BMSC therapy in stroke rats (Shen et al., 2007a). This report proved that BMSCs have an effect on scarring reduction and cell proliferation increase. In 2013, Chen, Jl. concluded that while the effect of multiple injections did not outperform single-injection therapy in terms of functional outcomes and histological evaluations, it significantly improved long-term functional outcomes following stroke (Shehadah et al., 2013). In 2014, in a review article published in *Progress in Neurobiology*, Chen, Jl. described the application of stem cells from various cell origins in stroke as well as the restorative mechanisms, distribution methods, and imaging methodologies, which also covered the difficulties in stem cell therapy converting to clinical applications (X. Liu et al., 2014). The aforementioned studies generally focus on the mechanisms and therapeutic benefits of stem cell treatment for stroke, indicating that the field is still in the development stage and that additional basic and clinical translational research are still required. Clearly, the achievements of Chen, Jl. have laid a theoretical and experimental basis for research on stem cells in stroke.

A reference is deemed to be co-cited if it is cited in a number of different publications, in which case the co-cited references could be viewed as the foundation of the field’s study. To determine the research foundation for stem cells in stroke, we selected 15 references with the largest number of co-citations for this bibliometric analysis. Among the top 15 co-cited publications, two were written by Caplan et al., the first of which was the most often mentioned study and was published in the *Journal of Cellular Biochemistry* in 2006. This study firstly showed the trophic of the MSC-secreted bioactive molecules and summarized the application of the MSC trophic effect in injured tissues (Caplan and Dennis, 2006). The biological mechanisms of the *in vivo* functionality of MSCs during development and aging were outlined in another study (Caplan, 2007). The second most co-cited study was written by Chamberlain, G. et al., in 2011 and published in *STEM CELLS*. Their discovery that the movement of MSCs from the circulation into tissues may be facilitated by chemokine receptors and adhesion molecules attracted interest in terms of the function of stem cells in immunological regulation. Of the top 15 co-cited references (Abrous, Koehl, and Le Moal, 2005; Ohab et al., 2006), two were about the neural regeneration function of stem cells, outlining the molecular mechanisms between stem cells and neurogenesis and suggesting that stem cell therapy may be a therapeutic strategy for nervous system diseases. Three references (Schmidt-Lucke et al., 2005; Falk, 2006; Dutta et al., 2012) demonstrated that stem cells can repair blood vessels and promote neoangiogenesis. Overall, the biological function, transplantation, components, and targeted delivery of stem cells are the main subjects of discussion in the top 15 co-cited references, which represent the research foundation of stem cells in stroke.

### 4.2 Hotspots and frontiers

Citation bursts refer to references that have received a lot of recent citations from other scholars and highlight emerging themes within a specific research field. In accordance with the key research topics of the references with the strongest citation bursts (Figure 7C), the potential mechanism of directed migration and neurogenesis of NSCs as well as the therapeutic effects of stem cells in stroke are currently the main areas of study for research on stem cells in stroke. In addition to references with citation bursts, keywords can facilitate swift capturing of the distribution and development of hotspots in the realm of research on stem cells in stroke. Combining the citation bursts and keywords, we prepare to divide the hotspots and frontiers into two main research areas, namely, the mechanistic research hotspots and the clinical research hotspots, to discuss the distribution of their respective hot spots according to Table 6 and Figures 8A, B.

### 4.3 The hotspot mechanisms of stem cells in stroke

#### 4.3.1 Stem cell and regeneration mechanism

As can be seen from Table 6, “neurogenesis,” “angiogenesis,” and “neural stem cells” are currently the focus of research in stem cell regeneration. Nerve and cerebrovascular regenerations are essential for recovery from stroke (Zhang et al., 2022). In 1992, Reynolds, Weiss, et al. isolated NSCs and fostered for the first time in the presence of epidermal growth factor, leading to large cell spheres they called “neurospheres” (Reynolds and Weiss, 1992). The discovery of NSCs provides a new promising therapy for nerve and vascular regenerations in stroke. Neuronal and glial cells originate from the common immature NSC, defined as self-renewing and multipotent cells that can differentiate into neurons, astrocytes, and oligodendrocytes (Reynolds and Weiss, 1992). NSCs were found to exist not only in the developing brain but also in the mature mammalian brain. Many studies have demonstrated that NSCs can replace lost neurons and restore connectivity in neuronal circuits, contributing to improved recovery from stroke and brain injury in rats (Daadi et al., 2009; Yokobori et al., 2019; Abeysinghe et al., 2015; Hou et al., 2017; G; Wang et al., 2020). Transplanted NSCs may prevent neuronal apoptosis, exert immunomodulatory effects both inside and outside the brain, and increase endogenous neuronal regeneration and angiogenesis (G.-L. Zhang, Zhu, and Wang, 2019; Horie, Hiu, and Nagata, 2015). Numerous studies have evaluated the therapeutic efficacy and safety of transplanted exogenous NSCs in preclinical animals with cerebral ischemic stroke (Daadi et al., 2009; Horie et al., 2011; Yokobori et al., 2019). However, due to the limited regeneration capacity of NSCs, the physiological environment is complex, which limits their repair effect. The current alternative approach to the use of NSCs is the use of inducible pluripotent stem cells or MSCs. Tobin and colleagues reported that both activated and naive MSCs induced complete behavioral recovery, reduced infarct volumes, and reduced microglial activation and IL-1β, TNF-α, and IL-6 levels in treated animals compared with vehicle-treated control stroke animals (Tobin et al., 2020). The angiopoietin expression and blood vessel density in ischemic brain tissue significantly increased after MSC transplantation (Zhang et al., 2022). MSC transplantation can promote neurogenesis mainly involving enhancement of endogenous neural cell proliferation and protection of newly grown cells from the pathogenic environment. A recent study demonstrated that MSC spheroid-loaded collagen hydrogels played a therapeutic role through three upregulated signals related to cell communication and upregulated the PI3K-Akt signaling pathway, which increased the expression of proteins related to neurogenesis and neuroprotection (He et al., 2021b). As MSCs can be used to promote the differentiation of NSCs into neurons by the production of different classes of trophic factors and anti-apoptotic molecules, future studies can focus on the development of MSC cell therapies associated with NSCs to facilitate nervous system recovery.

#### 4.3.2 Antioxidant and anti-inflammatory mechanism

Research on “oxidative stress” and “inflammation” have gradually become popular in recent years, suggesting that scholars have investigated stem cells into a deeper level. “Oxidative stress” and “inflammation” were among the top 25 keywords from 2020 to 2022 with the most citation spikes. Previous research demonstrated that oxidative stress and inflammation are two of the initial steps in the chain of events leading to cerebral ischemia injury, which disrupts various neuronal circuits (Rana and Singh, 2018; Chen H. et al., 2020; Chen S. et al., 2020). It has been commonly acknowledged that inflammation plays a major role in the development and course of the disease, as well as in recovery and wound healing following a stroke (Shekhar et al., 2018). Meanwhile, the potential of antioxidant therapies for stroke is suggested by the presence of induced oxidative modulatory pathway in the development of stroke (Pradeep et al., 2012). In recent years, it has been proven that stem cell therapy is a potentially successful treatment for inhibiting inflammation and oxidative stress following stroke (Lei et al., 2022). Researchers found that MSCs reduced the level of cellular oxidative stress and elevated the intracellular calcium and reactive oxygen species of neuronal cells when under stress from cerebral ischemia (K.-H. Chen et al., 2016; Alhazzani et al., 2018). In addition, emerging evidences have suggested that stem cells can increase the effectiveness of mitochondrial transfer to improve oxidative phosphorylation, lessen cellular oxidative stress levels and subsequently the brain damage cascade caused on by ischemic injury (Tseng et al., 2021; K; Liu et al., 2019). Based on the foregoing discussion, by lowering the degree of oxidative stress and transferring healthy mitochondria to damaged cells, stem cells engage their antioxidant ability in ischemic stroke. As for the inhibition of inflammation, recent research suggested that stem cells can regulate immune cell infiltration and polarization in ischemic brain to reduce neuroinflammation (Li et al., 2019; Yang et al., 2020). Interestingly, many studies reported no difference in or even worse efficacy of anti-inflammatory agents for stroke (Iosif et al., 2008; Tobin et al., 2014). However, some researchers used anti-inflammatory compounds to strengthen the anti-inflammatory effects of MSCs. For instance, some investigators found that MSCs from human umbilical cords that overexpress C–C motif chemokine ligand two or MSCs that have been activated with interferon-γ have a stronger anti-inflammatory ability in ischemic stroke compared with naive MSCs (Lee et al., 2020; Tobin et al., 2020). Collectively, oxidative stress and inflammation are both mechanisms of the main occurrence and development of stroke. Stem cells can fight oxidative damage, reduce inflammation, and alleviate aggravation of stroke. However, the mechanisms involves many pathophysiological processes and molecular pathways, which are needed to figure out, including the phenomenon caused by MSCs combined with anti-inflammatory agents, as described above. Therefore, in an attempt to understand the underlying mechanism, more research on mechanism is warranted.

#### 4.3.3 Stem cells and extracellular vesicles

“Mesenchymal stem cells” are among the current study hotspots in terms of stem cell type selection. Among several stem cell types, MSCs have attracted the attention of numerous researchers. MSCs can be simply extracted from the bone marrow (51%), umbilical cord (17%), and adipose tissue (11%) (Kabat et al., 2020). MSC therapy has been proven to reduce inflammation, encourage neurogenesis, and inhibit angiogenesis and apoptosis to improve neuronal defects, neural network reconstruction, and neurological functions (Zhu et al., 2019). Cui J. et al. found that MSCs can reduce neurological impairments and enhance axonal regeneration in rats with stroke (Cui et al., 2017). Furthermore, MSCs exert angiogenic effects based on secreted angiogenic factors by significantly enhancing vitality, motility, and network formation (König et al., 2015). The fact that neuroinflammation accelerated the development of brain injury is widely accepted. The method for controlling immune response may thereby decrease brain damage. Studies have demonstrated that stem cells can facilitate nerve repair either by boosting the protective effects of anti-inflammatory cytokines or performing immunomodulatory functions, such as neutrophil and microglia regulation (Jingli et al., 2022). For instance, following a stroke, MSCs can suppress microglial activation by upregulating growth factors and hypoxia-inducible factor-1-alpha while downregulating proinflammatory cytokines and chemokines (Yan et al., 2013). Current studies have verified that the neuroprotection of MSCs in stroke. Therefore, enhancing the therapeutic benefits and immunomodulatory capabilities of MSC may provide researchers with potential targets for their future studies.

From the stem cell derivatives used in the treatment of stroke, our data (Figure 8B) indicated that the latest research hotspots from 2019 to 2022 were “exosomes” and “extracellular vesicle.” Extracellular vesicles, which can transport a variety of cargos, including lipids, nucleic acids, and proteins, and are released from the cell surface into body fluid, help cells communicate with one another (Allan et al., 2020). One of the most currently appealing subcategories of extracellular vesicles is exosome (Lawson et al., 2016). In recent years, studies have suggested restrictions and potential hazards with stem cell therapy, such as tumorigenicity and the inability to successfully penetrate the blood-brain barrier (BBB) (Lukomska et al., 2019). Researchers also found that the use of exosomes generated from stem cells could be an alternative strategy to stem cell (Venkat, Chopp, and Chen, 2018; Cai et al., 2020). The nanoscale characteristics of exosomes allow themselves to efficiently disperse throughout the body and pass across the BBB. Furthermore, it can imitate the ability of stem cells and other supporting cells to regenerate. Stem cells release exosomes to communicate with other cells, when they detect alterations in the microenvironment, such as “inflammation” or “oxidative stress.” Exosomes have been demonstrated to be useful for the regulation of post-stroke inflammation, neurovascular remodeling, angiogenesis, neurogenesis, synaptic plasticity, and apoptosis and autophagy control (Seyedaghamiri et al., 2022). According to some authors, exosomes can be used as natural biomarkers to gage the seriousness of clinical manifestations of neurological diseases (Hong et al., 2019).

#### 4.3.4 Clinical application prospect of stem cells in stroke

Basic science and animal models have laid the groundwork for advancing stem cell therapy for stroke in clinical setting. NSC transplantation has been performed mainly as a treatment for chronic phase of post-stroke. In a PISCES one clinical trial (Kalladka et al., 2016), the NSC transplantation to the patients within 6 months to 5 years after stroke showed that the embedded delivery of the NSC line CTX0E03 was safe and suggested improved neurological function. However, the study was conducted on only 11 men; thus, further study is needed that includes female patients and larger patient populations. In another PISCES two study that included adults aged over 40 years with significant upper-limb motor deficit 2–13 months following ischemic stroke, 23 patients underwent CTX cell transplantation, and their upper-limb functions improved at 3, 6, and 12 months (Muir et al., 2020). Subsequently, the PISCES-3 study recruited approximately 130 patients with moderate to severe functional disability from 6 to 24 months after the stroke. The primary outcome was an improvement in the modified Rankin scale (mRS) score at 6 months following surgery (Wechsler et al., 2018). However, due to the ethical issues related to the harvesting of fetal NSCs, as well as the limited number of donors, very few trials used NSCs. In addition, the clinical use of NSCs has several disadvantages, such as immunogenicity and the possibility of rejection of allogeneic human NSCs, which limits its application.

A large number of preclinical data have proven the feasibility of MSCs in the treatment of stroke, and clinical administration of stem cell therapy is expected. Most of the clinical trials have evaluated the efficacy and safety of MSCs for the treatment of stroke. MSCs, especially bone marrow-derived ones, are most widely used in clinical trials. In a randomized study of 30 patients with severe stroke, Bang et al. reported that MSCs improved the mRS and Barthel index scores within 1 year following stroke and exerted no adverse cell-related, serological, or imaging-defined effects (Bang et al., 2005). Honmou et al. reported that there were no major side events after the intravenous infusion of autologous BMSCs expanded in human serum into 12 participants 36–133 days post-stroke (Honmou et al., 2011). Overwhelming evidence supports the safety of the approach, although data on its efficacy are scarce or indicate only a transient improvement (Nistor-Cseppentö et al., 2022). Majority of the adverse events in these clinical trials were minor (Kvistad et al., 2022). For example, two minor side effects in a clinical trial (n = 57) using MSCs to treat stroke may be linked to venous internal position stimulation and urinary tract infection (Levy et al., 2019). However, some animal studies have demonstrated that MSCs may increase the risk of autoimmune disease and the onset of tumors (Djouad et al., 2003). This prompted the researchers to look for some countermeasures. Furthermore, most of the relevant clinical trials included only a few patients (n < 100), and large multicenter randomized controlled trials are absent; thus, further research is warranted to determine the efficacy and security of MSCs for stroke treatment and also to identify the optimal cell concentration, time, patient selection criteria (age, stroke subtype, and damage area), and combination therapy for routine clinical uses.

Most of the studies on exosomes have focused on animal and *in vitro* experiments. According to available information, a few clinical studies have used exosomes for stroke treatment. As of August 2022, only three clinical trials involving stroke patients were available on the public clinical trial database (https://clinicaltrials.gov/). One study evaluated improvement in patients suffering from acute ischemic stroke who were administered with allogenic MSC-derived exosomes. In a different study, the diagnostic value of blood extracellular vesicles was investigated in stroke patients undergoing rehabilitation. The final study aimed to determine how acupuncture-induced exosomes may help treat post-stroke dementia. In addition, a pilot randomized clinical trial suggested that local injection of exosomes produced by allogenic placenta MSCs is safe after a malignant middle cerebral artery infarction (Dehghani et al., 2022). These data suggest that exosome therapy is a novel promising strategy for stroke in clinical translational application. In conclusion, although the therapeutic effect, biosafety, kinetics, and biodistribution of exosomes still need to be thoroughly investigated, their capacity for regeneration and repair provides new possibilities for the treatment of stroke.

### 4.3 Advantages and limitations

This study has several advantages. First, based on research published from 2004 to 2022, this bibliometric analysis is the first investigation of patterns and contentious topics relating to stem cells in stroke. Second, we used three bibliometric methods simultaneously for the survey and analysis in this study, which significantly increased the likelihood that our data analysis process is impartial; VOSviewer and CiteSpace were extensively used in this study (C. Chen et al., 2012). This study also involved a thorough analysis of the number and growth tendency of annual publications; relationships among journals, authors, nations, and institutions; and various references, citations, and keywords.

This study also has limitations that need to be acknowledged. First, because this study only included articles in English, non-English writings may have been underrepresented. Second, only data obtained from the WoSCC database were used in this study; therefore, some pertinent studies from other databases may have been missed. Furthermore, insufficient data prevented full inclusion of articles in 2022.

## 5 Conclusion

This analysis may help researchers in identifying new trends and research hotspots for stem cells in stroke in the period of 2004–2022. The steadily increasing number of publications indicates that research on stem cells in stroke is becoming more and more important to academics worldwide. The top 3 countries with the most number of publications were China, the USA, and Japan. The journal, organization, and author with the most influence on were *Cell Transplantation*, Florida State University, and Chopp, M. respectively. The keywords that highlight recent hot topics about research on stem cells in stroke were “neurogenesis,” “angiogenesis,” “mesenchymal stem cells,” “oxidative stress,” “inflammation,” “exosomes,” and “extracellular vesicles,” which will probably become promising in the future. Notably, stem cells have numerous positive effects, such as neuroprotection, enhanced angiogenesis and neurogenesis, and diminished inflammatory and immunological responses; however, the main mechanisms for mitigating the damage caused by stroke are still unknown. Clinical challenges may include complicating factors, such as the effect of age, stroke subtype, and stroke severity, all of which can affect the efficacy of stem cell therapy. In the future, to successfully create a therapeutic scheme, all of complicating factors must be carefully taken into account.

## Data Availability

The original contributions presented in the study are included in the article/Supplementary Material, further inquiries can be directed to the corresponding authors.
