# Supplementary material for: Research hotspots and frotiers of stem cells in stroke: A bibliometric analysis from 2004 to 2022
Source: Front Pharmacol. 2023 Mar 3;14:1111815. doi: 10.3389/fphar.2023.1111815 (PMC10020355; doi:10.3389/fphar.2023.1111815)
Supplement: Supplementary file 1 [file Table1.DOCX]

| **Supplementary Table 1 Advantages and Disadvantages of the Top 25 Articles with the Strongest Citation Bursts** | | |
| --- | --- | --- |
| **References** | **Advantage** | **Disadvantage** |
| Arvidsson A, 2002, NAT MED, V8, P963 | The article provides the first evidence that the adult brain can use neuronal replacement from endogenous precursors to repair itself after stroke. | The signaling molecules attracting the new neurons had not been explored in this article. |
| Parent JM, 2002, ANN NEUROL, V52, P802 | The article indicates that focal ischemia in the adult rat increases forebrain SVZ neurogenesis and leads to the generation of new neurons with appropriate regional specificity in the periinfarct neostriatum. | The molecular clues underlying ischemia-induced neurogenesis and altered neuroblast migration have not been explored. |
| Nakatomi H, 2022, CELL, V110, P429 | The article shows that following ischemic brain injury, adult neural progenitors can be stimulated in situ by intraventricular infusion of growth factors to replace CA1 pyramidal neurons. These newly generated CA1 neurons form functional synapses and are integrated into the existing brain circuitry. In parallel to the massive induction of new hippocampal neurons, the growth factor treatment ameliorates deficits in hippocampal-dependent spatial cognitive functions in ischemic animals. | The relationship between regenerated hippocampal neurons and cognitive recovery was not elucidated. |
| Jin KL, 2001, P NATL ACAD SCI USA, V98, P4710 | The article indicates that focal cerebral ischemia promotes neurogenesis in both SGZ and SVZ and provide a model for investigating the mechanisms that underlie this phenomenon and its functional consequences. Besides, they finding is that focal ischemia increases incorporation of BrdUrd, a marker for DNA replication and a surrogate for cell division, in both regions. | It is not clear that how a neurogenic signal is transmitted from a site of stimulation or injury to neuroproliferative zones of the brain. It is uncertain whether adequate numbers of cells can be generated to repopulate a large destructive lesion such as an infarct, especially because many newly generated neurons undergo programmed cell death. |
| Jin K, 2003, MOL CEKK NEUROSCI, V24, P171 | The article maps the location of BrdU-labeled cells and cells expressing markers of neuronal lineage for up to 2 weeks following focal cerebral ischemia, induced by transient occlusion of the middle cerebral artery in rats. They report that doublecortin-expressing cells from the rostral subventricular zone, migrated into the ischemic penumbra of the adjacent striatum and, via the rostral migratory stream and along the path of the embryonic lateral cortical stream, into the penumbra of ischemic cerebral cortex and they could exert a role in functional recovery. | In this research, where migration rate is determined based on labeling with BrdU or thymidine, the time required for cells to enter S phase of the cell cycle and incorporate these markers could cause the rate to be underestimated. |
| Taguchi A, 2004, J CLIN INVEST, V114, P330 | The results of the article demonstrate that systemic administration of human CD34+ cells to immunocompromised mice subjected to stroke 48 hours earlier accelerates neovascularization of the ischemic zone. Their results provide the first direct link between vasculogenesis and neurogenesis in the repair of ischemic brain lesions. | The possible molecular mechanism of CD34+ enhancement of neovascularization was not explored in this study. |
| Zhang RL, 2004, J CEREBR BLOOD F MET, V24, P441 | We used an*in vitro*model of ischemia and present several lines of evidence that Gap-junctional communication (GJC) promotes cell death in organotypic hippocampal slices over the 48-hour period after hypoxic–hypoglycemic insult. | The possible role of GJC is promoting or decreasing injury that is still debated. |
| Wang L, 2004, STOKE, V35, P1732 | The article indicate that treatment with rhEPO beginning 24 hours after stroke significantly improved functional recovery and concomitantly enhanced angiogenesis and neurogenesis. Furthermore, they indicate that EPO induces angiogenesis via regulation of VEGF. | They have not demonstrated that neuroblasts in the ischemic boundary regions integrate into the cerebral architecture and have mature neuronal electrophysiological properties, the data associating neurogenesis with functional recovery should be interpreted with caution. |
| Shyu WC, 2004, CIRCULATION, V110, P1847 | In this study, they demonstrate that subcutaneous injections of G-CSF, starting 1 day after cerebral ischemia and continuing for up to 5 days, enhance neural repair in rats suffering from cerebral ischemia. Infarction volume was markedly reduced, and there was also significant recovery of neurological dysfunction. | The mechanism by which HSC recovery of neurological dysfunction has not been investigated. |
| Bang OY, 2005, ANN NEUROL, V57, P874 | In this study, we evaluated the long-term prognosis and neuroradiological features after intravenous injection of autologous MSCs in patients with cerebral infarcts within the middle cerebral artery (MCA) territory and with severe neurological deficits. | The optimal time at which MSC infusion should occur after a stroke is unknown. The study had a small sample size, which may have made it difficult to produce a knot that illustrates effectiveness. Double-blind studies with larger cohorts are needed to reach a definitive conclusion regarding the efficacy of MSC therapy. In addition, further studies are needed to determine which stroke patients should undergo transplantation, because the location, severity, and chronicity of the stroke and the adequacy of blood supply will likely affect the efficacy of MSC therapy. |
| Imitola J, P NATL ACAD SCI USA, V101, P18117 | Their data provide not only a working model for one mechanism underlying innate regenerative programs but also a unifying explanation as to why NSCs appear to behave so similarly when confronted with pathologies of disparate etiologies. Their data einforce the pivotal role played by the SDF-1α/CXCR4 pathway and likely other inflammation-associated mechanisms [including, for example, microglia in the migration of NSCs toward regions of brain injury and degeneration. | Survival of HNSCS migrating to the inflamed area was not verified. |
| Thored P, 2006, STEM CELLS, V24, P739 | The data provide the first evidence that the adult brain responds to a stroke by long-lasting generation of neurons from its own NSCs.  They also provide the first evidence that SDF-1α/CXCR4 signaling regulates the directed migration of new striatal neurons generated from endogenous NSCs toward the ischemic damage. Their finding that stroke-induced neurogenesis is persistent has several implications. | They didn’t prove a causal relationship between neurogenesis and behavioral improvement after stroke. The use of specific CXC4 blockers did not completely inhibit the migration of new neurons, which suggests that other mechanisms also direct the new neurons to the damaged area. |
| Yamashita T, 2006, J NEUROSCl | The article reveals the subventricular zone to be the principal source of the neuroblasts that form chain-like structures migrate laterally toward the injured striatal regions and differentiate into mature neurons. | it remains unclear what types of neurons are generated. Thus, it will be necessary to add appropriate interventions to enhance the proliferation, survival, and/or neuronal maturation of the subventricular zone cells and their progeny. |
| Ohab JJ,2006, J NEUROSCl | The article is the first causal link between endogenous angiogenesis and neurogenesis after stroke. | The article needed more behavioral experiments to verify behavioral recovery after stroke. |
| Lee JS, 2010, STEM CELLS, | The study hints the  long-term safety of i.v. autologous ex  vivo cultured MSCs infusion in patients with acute ischemic stroke. In addition, they evaluated factors associated with the response to MSC therapy. | The article is not conclusive because of the small sample size as well as his study was not double  blinded, and they do not allow elucidation of the direct cause and effect relationships between SDF-1a levels and MSC effects. |
| Honmou O, 2011,BRAIN | The article demonstrate the feasibility of studies on administration of human serum-treated autologous human MSCs, suggest that these studies can be carried out safely and underscore the need to assess functional outcome at early, as well as late, times after cell administration. | The study was unblinded and does not exclude placebo effects or recovery as a result of the natural history of stroke. |
| Savitz SI. 2011  ANN NEUROL | this study is the first clinical trial to address the feasibility and safety of a bone marrow harvest in acute stroke patients followed by autologous IV reinfusion of purified MNCs within 24 to 72 hours of  symptom onset. | This study had a number of other limitations and many of the patients were young, had cryptogenic strokes, and had few vascular risk factors. |
| Prasad K, 2014, STROKE | The article is the first and the largest randomized controlled trial comparing intravenous infusion of autologous BMSCs and control in patients with subacute ischemic stroke. | The article includes lack of blinding of patients or physicians and their population had too severe stroke (mean infarct volume 99.3) to benefit from cell therapy. |
| Doeppner TR,2015,STEM CELL TRANSL MED | The study systematically compared the effects after MSC-EV and MSC administration on neurological recovery and brain remodeling in a mouse model of transient focal cerebral ischemia. | The study the used EV fractions in were derived from nonstimulated MSC cultures and albeit other delivery routes were not studied in the work. |
| Steinberg GK, 2016,STROKE, | This is the first reported intracerebral stem cell transplant study for stroke in North America,evaluated the safety and clinical outcomes of the stereotactic placement of SB623 cells at the margin of the stroke in patients with chronic motor deficits >6 months after their initial stroke. | This study is a small-scale, open-label, dose-escalation, Phase 1/2a trial and is therefore limited by its nonrandomized, uncontrolled design and small number of patients. |
| Kalladka D, 2016,LANCET, | This “first-in-man” study offers preliminary data on the feasibility, tolerability and cell-related safety of stereotactic intra-cerebral injection of the genetically modified human neural stem cell line CTX0E03-DP in patients with chronic ischaemic stroke. | The article has open label design, lack of control subjects and a small sample size by design limits the number of patients being exposed to each dose level the . |
| Hess DC,2017,LANCET NEUROL | Thrie early time window of 24–36 h with intravenous delivery is complementary to other stereotactic intracerebral transplantation approachesthat target stroke . | This trial included a relatively small sample size and their measurement of white blood cells and inflammatory biomarkers was limited to measurements in the serum . |
| Xin HQ,2017,STROKE | This study, for the first time, employs exosomes engineered with a specific miRNA cluster gene to treat stroke, and demonstrate that these tailored exosomes provide an increased therapeutic effect on neurological recovery compared to the functional benefits derived from treatment with naive exosomes. | This study lacks on the quantification of mRNA levels of PTEN in neurons and connective tissue growth factor (CTGF) and Tsp1 in astrocytes. |
