# Supplementary figures and images for: Research hotspots and frotiers of stem cells in stroke: A bibliometric analysis from 2004 to 2022
Source: Front Pharmacol. 2023 Mar 3;14:1111815. doi: 10.3389/fphar.2023.1111815 (PMC10020355; doi:10.3389/fphar.2023.1111815)

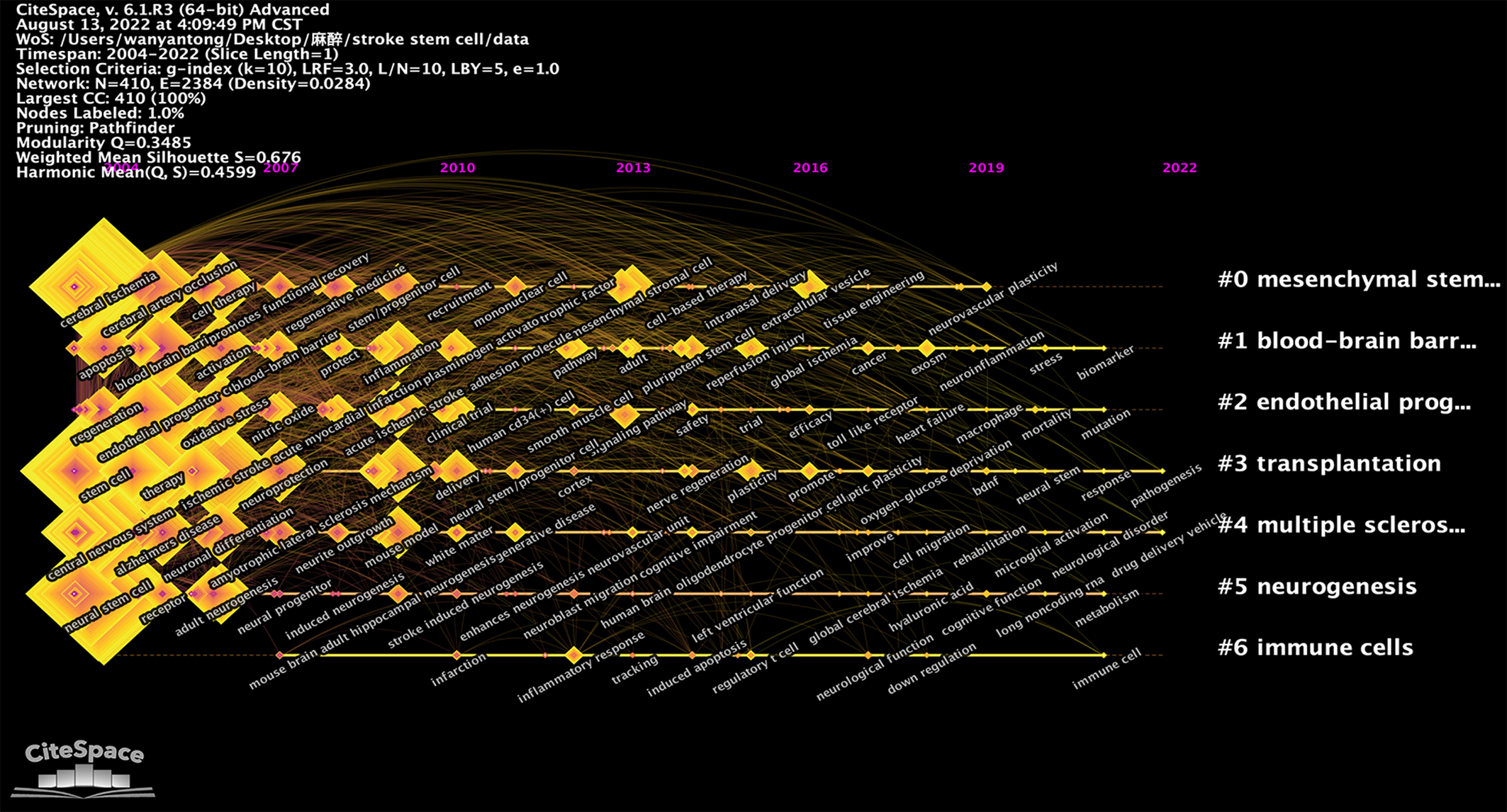

Supplement: Supplementary file 2 [file Image2.TIF]

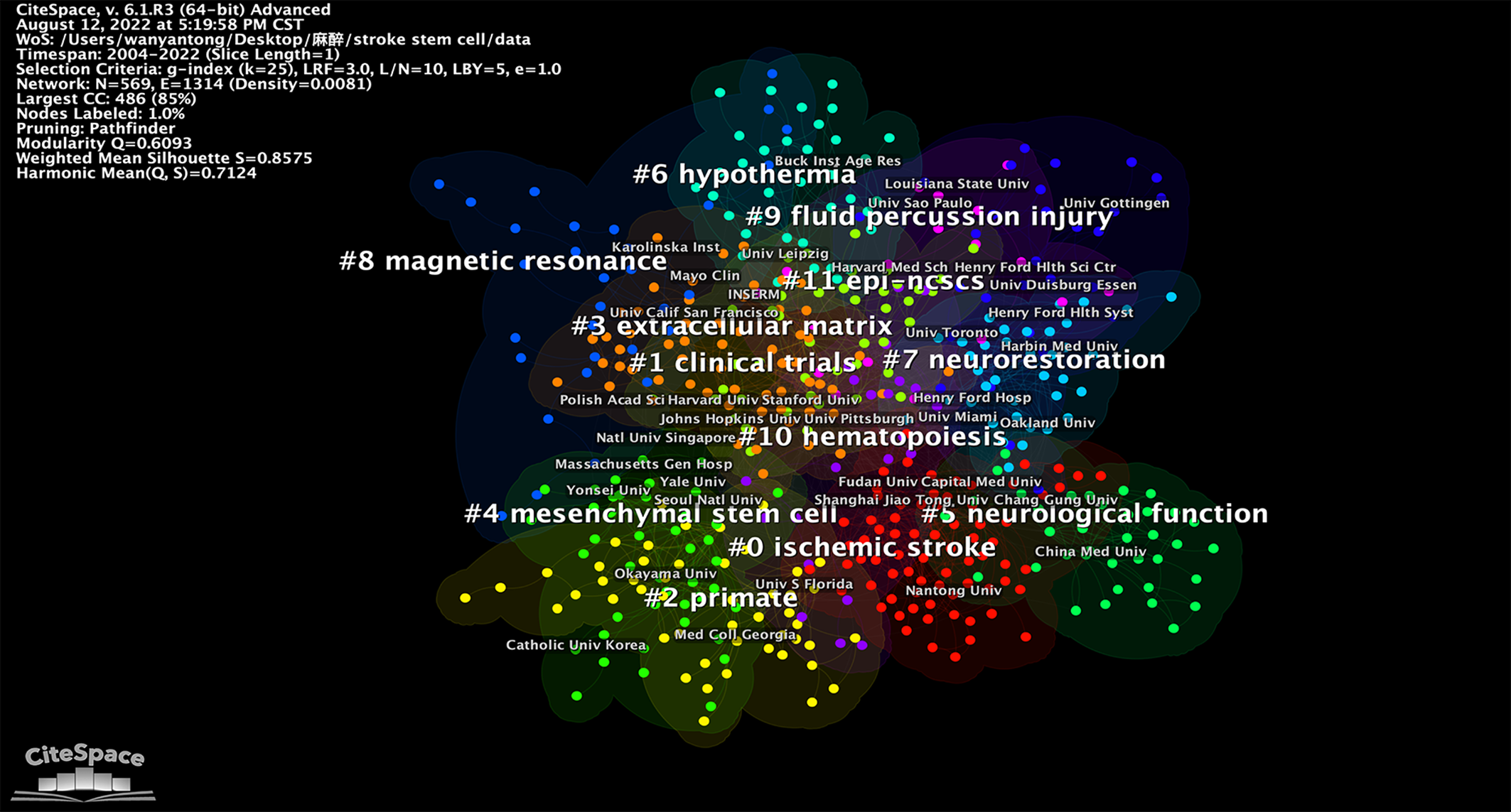

Supplement: Supplementary file 3 [file Image1.TIF]
